# Supplementary material for: Uncovering the basis of protein-protein interaction specificity with a combinatorially complete library
Source: eLife. 2020 Oct 27;9:e60924. doi: 10.7554/eLife.60924 (PMC7669267; doi:10.7554/eLife.60924)
Supplement: Supplementary file 1. — Table S1. Crystallographic data and refinement statistics Table S2. Plasmids created in this study Table S3. Oligonucleotides used in this study [file elife-60924-supp1.docx]

# Table S1. Crystallographic data and refinement statistics^&^

|  | ParD2*-ParE2  (PDB: 6X0A) |
| --- | --- |
| Wavelength (Å) | 0.9792 |
| Resolution range (Å) | 29.81 - 2.9 (3.004 - 2.9) |
| Space group | P 32 |
| Unit cell | 148.54 148.54 195.27 90 90 120 |
| Total reflections | 514447 |
| Unique reflections | 105516 |
| Completeness (%) | 98.62 (93.14) |
| Mean I/sigma(I) | 10.3 |
| Wilson B-factor | 88.60 |
| R-merge | 0.117 (0.914) |
| R-meas | 0.131 (1.040) |
| R-pim | 0.059 (0.486) |
| CC1/2 | 0.996 (0.745) |
| Reflections used in refinement | 105335 (9955) |
| Reflections used for R-free | 1998 (190) |
| R-work | 0.2427 (0.3563) |
| R-free | 0.2831 (0.4076) |
| RMS (bonds) | 0.006 |
| RMS (angles) | 0.86 |
| Ramachandran favored (%) | 97.23 |
| Ramachandran outliers (%) | 0.13 |
| Rotamer outliers (%) | 1.02 |
| Clashscore | 19.89 |
| Average B-factor | 116.53 |

^&^Statistics for the highest resolution shell are shown in parentheses.

# Table S2: Plasmids created in this study^&^

|  | **plasmid** | **insert** |
| --- | --- | --- |
| ML3295 | pEXT20 | ParD2 |
| ML3296 | pEXT20 | ParD3 |
| ML3297 | pETDuet-1 | ParD2 his-ParE2 |
| ML3298 | pEXT20 | ParD3 IKE |
| ML3299 | pEXT20 | ParD3 DLE |
| ML3300 | pEXT20 | ParD3 DKK |
| ML3301 | pEXT20 | ParD3 ILK |
| ML3302 | pBAD33 | ParE3 |
| ML3303 | pBAD33 | ParE2 |
| ML3304 | pEXT20 | Library template* |
| ML3305 | pETDuet-1 | ParD3-ParD2 chimera his-ParE2 (see Figure S9) |
| ML3306 | pEXT20 | ParD3 WLK |
| ML3307 | pEXT20 | ParD3 MIN |
| ML3308 | pEXT20 | ParD3 EVW |
| ML3309 | pEXT20 | ParD3 DYQ |
| ML3310 | pEXT20 | ParD3 library |

^&^*Escherichia coli* strains used in this study are TOP10 (ThermoFisher cat #: C404050) and T7 express (NEB cat #: C2566I). ParD3 variants indicate amino acid at positions 61, 64, and 80, respectively.

ParD3 wt MANVEKMSVAVTPQQAAVMREAVEAGEYATASEIVREAVRDWLAKRELRHDDIRRLRQLWDEGKASGRPEPVDFDALRKEARQKLTEVPPNGR*

|||||||||||||||||||||||||||||||||||||||||||||||||||||||||||| ||

*Library template MANVEKMSVAVTPQQAAVMREAVEAGEYATASEIVREAVRDWLAKRELRHDDIRRLRQLWIEGERRLGKS*

# Table S3: Oligonucleotides used in this study

| **Primer** | **Name** | **Sequence (5’** → **3’)** | **Use** |
| --- | --- | --- | --- |
| MTL01 | L: sequencing primer for pEXT20 inserts | ccgacatcataacggttctggc | Cloning |
| MTL02 | R: sequencing primer for pEXT20 inserts | TTTCTTCTCTCATCCGCCAAAACAG | Cloning |
| MTL03 | L: sequencing primer for pBad33 inserts | ttgcacggcgtcacactttgct | Cloning |
| MTL04 | R: sequencing primer for pBad33 inserts | tgggaccaccgcgctactgc | Cloning |
| MTL05 | L: sequencing primer for expression plasmids | GGATCTCGACGCTCTCCCT | Cloning |
| MTL06 | R: sequencing primer for expression plasmids (standard T7 terminal primer) | gctagttattgctcagcgg | Cloning |
| MTL07 | L: oligo to make triple mutant ParD3 library | aacgcGTCGAAATCCACGGGCTCCGGTCTCCCGCTTGCmnnGCCTTCmnnCCAGAGCTGCCTCAGCCGGCGGATA | High-throughput sequencing |
| MTL08 | R: oligo to make triple mutant ParD3 library | GCAAGCGGGAGACCGGAGCCCGTGGATTTCGACGCGTTGCGAAAGnnkGCTCGGCAAAAGCTGACGGAAGTCC | High-throughput sequencing |
| MTL09 | L: Illumina primer for sequencing ParD3; adds NNNN spacer | AATGATACGGCGACCACCGAGATCTACACTCTTTCCCTACACGACGCTCTTCCGATCTNNNNATGGCAAACGTGGAAAAAATGAGC | High-throughput sequencing |
| MTL10 | L: Illumina primer for sequencing ParD3; adds NNNNN spacer | AATGATACGGCGACCACCGAGATCTACACTCTTTCCCTACACGACGCTCTTCCGATCTNNNNNATGGCAAACGTGGAAAAAATGAGC | High-throughput sequencing |
| MTL11 | L: Illumina primer for sequencing ParD3; adds NNNNNN spacer | AATGATACGGCGACCACCGAGATCTACACTCTTTCCCTACACGACGCTCTTCCGATCTNNNNNNATGGCAAACGTGGAAAAAATGAGC | High-throughput sequencing |
| MTL12 | R: Illumina primer for sequencing MO ParD3 wt; adds barcode gcacatct | CAAGCAGAAGACGGCATACGAGATgcacatctGTGACTGGAGTTCAGACGTGTGCTCTTCCGATCTCTAACGGCCATTCGGCGGGACTTC | High-throughput sequencing |
| MTL13 | R: Illumina primer for sequencing MO ParD3 wt; adds barcode agcaattc | CAAGCAGAAGACGGCATACGAGATagcaattcGTGACTGGAGTTCAGACGTGTGCTCTTCCGATCTCTAACGGCCATTCGGCGGGACTTC | High-throughput sequencing |
| MTL14 | R: Illumina primer for sequencing MO ParD3 wt; adds barcode aaggatgt | CAAGCAGAAGACGGCATACGAGATaaggatgtGTGACTGGAGTTCAGACGTGTGCTCTTCCGATCTCTAACGGCCATTCGGCGGGACTTC | High-throughput sequencing |
| MTL15 | R: Illumina primer for sequencing MO ParD3 wt; adds barcode catgctta | CAAGCAGAAGACGGCATACGAGATcatgcttaGTGACTGGAGTTCAGACGTGTGCTCTTCCGATCTCTAACGGCCATTCGGCGGGACTTC | High-throughput sequencing |
| MTL16 | R: Illumina primer for sequencing MO ParD3 wt; adds barcode aacttgac | CAAGCAGAAGACGGCATACGAGATaacttgacGTGACTGGAGTTCAGACGTGTGCTCTTCCGATCTCTAACGGCCATTCGGCGGGACTTC | High-throughput sequencing |
| MTL17 | R: Illumina primer for sequencing MO ParD3 wt; adds barcode tcggaatg | CAAGCAGAAGACGGCATACGAGATtcggaatgGTGACTGGAGTTCAGACGTGTGCTCTTCCGATCTCTAACGGCCATTCGGCGGGACTTC | High-throughput sequencing |
| MTL18 | R: Illumina primer for sequencing MO ParD3 wt; adds barcode aggatcta | CAAGCAGAAGACGGCATACGAGATaggatctaGTGACTGGAGTTCAGACGTGTGCTCTTCCGATCTCTAACGGCCATTCGGCGGGACTTC | High-throughput sequencing |
| MTL19 | R: Illumina primer for sequencing MO ParD3 wt; adds barcode cctatgcc | CAAGCAGAAGACGGCATACGAGATcctatgccGTGACTGGAGTTCAGACGTGTGCTCTTCCGATCTCTAACGGCCATTCGGCGGGACTTC | High-throughput sequencing |
